# Sanger sequencing is no longer always necessary based on a single-center validation of 1109 NGS variants in 825 clinical exomes

**DOI:** 10.1038/s41598-021-85182-w

**Published:** 2021-03-11

**Authors:** A. Arteche-López, A. Ávila-Fernández, R. Romero, R. Riveiro-Álvarez, M. A. López-Martínez, A. Giménez-Pardo, C. Vélez-Monsalve, J. Gallego-Merlo, I. García-Vara, Berta Almoguera, A. Bustamante-Aragonés, F. Blanco-Kelly, S. Tahsin-Swafiri, E. Rodríguez-Pinilla, P. Minguez, I. Lorda, M. J. Trujillo-Tiebas, C. Ayuso

**Affiliations:** 1 Department of Genetics, Health Research Institute–Jimenez Diaz Foundation University Hospital (IIS-FJD), Avda. Reyes Católicos 2, 28040 Madrid, Spain; 2 Department of Genetics, University Hospital, 12 de Octubre, Madrid, Spain

**Keywords:** Genetic testing, Molecular medicine

## Abstract

Despite the improved accuracy of next-generation sequencing (NGS), it is widely accepted that variants need to be validated using Sanger sequencing before reporting. Validation of all NGS variants considerably increases the turnaround time and costs of clinical diagnosis. We comprehensively assessed this need in 1109 variants from 825 clinical exomes, the largest sample set to date assessed using Illumina chemistry reported. With a concordance of 100%, we conclude that Sanger sequencing can be very useful as an internal quality control, but not so much as a verification method for high-quality single-nucleotide and small insertion/deletions variants. Laboratories might validate and establish their own thresholds before discontinuing Sanger confirmation studies. We also expand and validate 23 copy number variations detected by exome sequencing in 20 samples, observing a concordance of 95.65% (22/23).

## Introduction

Next-generation sequencing (NGS) enables multiple genes to be analyzed simultaneously and cost-effectively, thus making it a widely applied and useful tool for the diagnosis of rare genetic disorders^1–3^. However, it is widely accepted that NGS variants need to be validated with the gold standard Sanger sequencing technique prior to reporting, even though both the costs and turnaround time of this approach are considerable. Several studies have reported that the validation of high-quality NGS variants adds little value, although most have been performed with targeted gene panels and small sample sets^4–9^. To our knowledge, only 3 studies have validated exome data^10,11^, including the recent extended validation of 845 variants in 383 genes from 342 whole exomes^12^.

Here, we present our experience in a single-center study in which we extensively assessed the need to further validate all NGS variants with an alternative technique in 1109 variants of 425 genes from 825 clinical exome samples. We also demonstrate the importance of appropriate internal quality controls to detect inevitable human errors and thus improve the reliability of laboratory results, and expand the validation of copy number variations (CNVs) detected by clinical exome panels.

## Methods

### Patients and samples

All patients (N = 825) included in this study were referred to the Clinical Genetics Service of Hospital Universitario Fundación Jiménez Díaz (HU-FJD) with the suspicion of a genetic disease. Written informed consent was obtained from each participant in accordance with institutional requirements. The study was reviewed and approved by the Research Ethics Committee of HU-FJD and fulfilled the principles of the Declaration of Helsinki.

Whole peripheral blood samples were collected from probands. For the analysis of prenatal cases, amniotic fluid or chorionic villus samples were collected. Genomic DNA was extracted following standard procedures.

### Clinical exome panel sequencing and data analysis

Clinical exome panels were sequenced consecutively using 2 different technologies, namely, the TruSight One Sequencing panel (TSO; *Illumina In, San Diego, CA, USA*) (N = 304) followed by the Clinical Exome Solution panel (CES; *Sophia Genetics*) (N = 521). These panels capture, respectively, 4813 and 4900 genes related to inherited diseases. More than 95% of the captured genes overlapped in both approaches.

Paired-end sequencing (2 × 150 bp) was carried out on a NextSeq 500 (-Illumina-). Bioinformatics analysis for the detection of single-nucleotide variants (SNVs) and small insertions/deletions (indels) in TSO samples was performed using the Burrows-Wheeler alignment (BWA) Enrichment App, which provides industry-standard alignment. Variant calling was based on the Genome Analysis Toolkit (GATK). An in-house Sophia Genetics pipeline was used for the analysis of the CES samples.

Detection of CNVs was only possible in CES samples and was performed using an in-house Sophia Genetics method based on a hidden Markov model. Specifically, the method evaluates the coverage levels of the target regions across all samples within the same sequencing run. The coverage is normalized by sample and target region, thus enabling CNV calling. A minimum of 8 samples per run is required.

Data was analyzed using a large number of virtual panels that included a wide variety of genes associated with genetic conditions, such as inherited retinal dystrophies, neuropathy, deafness, skeletal dysplasia, ataxia, myopathy, intellectual disability, and kidney disease. Variants were interpreted and classified by a team of experienced molecular geneticists following the criteria of the American College of Medical Genetics and Genomics^13^. We applied the Interactive Genomics Browser to visualize pathogenic variants, likely pathogenic variants, and variants of uncertain clinical significance in disease-related dominant genes or in recessive genes when found together with another pathogenic variant, likely pathogenic variant, or variant of uncertain significance. Variants were further validated using Sanger sequencing prior to reporting.

### Quality metrics

High-quality SNVs and indel variants were defined as (1) FILTER = PASS, (2) QUAL ≥ 100, (3) Depth coverage ≥ 20X, and (4) Variant fraction ≥ 20%. Variants that did not fulfil these requirements were considered low-quality variants, with the exception of 22 related-disease variants classified as pathogenic or likely pathogenic according the ACMG criteria that strictly and although close to the established threshold, met at least two of these four requirements.

The filter PASS depends on the pipeline used and is therefore different in TSO and CES samples. According to the manufacturer’s recommendations, the metrics used to filter out SNVs and indels in TSO and CES samples were, respectively, “LowGQ;LowGQX;LowMQ;LowQD;SB” and “homopolymer_region;off_target;low_coverage;low_variant_fraction”. Once again, according to the manufacturer’s recommendations, the CNV analysis was based on the following metrics: “coverage uniformity; median absolute deviation of read depth and off-target rate of reads”. More information is available under request.

### Validation of SNVs and indels detected by NGS

Mixed samples or samples affected by laboratory contamination were defined as “misleading samples”. Given that they were caused by pre-analytical but not technical errors, we did not take them into consideration for the validation analysis. Low-quality variants were also excluded, as they did not meet the criteria for discontinuing Sanger sequencing.

High-quality variants were further validated. With the exception of 217 outsourced variants (170 samples), all high-quality SNVs and indel variants were bi-directionally confirmed by Sanger sequencing in our laboratory following standard procedures. Primers were designed manually or by using the ExonPrimer in silico tool. All primers were always checked in the *SNPchecker* program to avoid common SNPs within the primer design. They were also checked in the In-silico PCR tool of the UCSC Genome Browser to confirm that they were specific for the region to be amplified.

### Validation of CNVs detected by NGS

All CNVs detected in CES samples were confirmed in our laboratory either by multiplex ligation-dependent probe amplification (MLPA) (N = 16) or by comparative genomic hybridization (CGH) array (60 k or 400 k custom array) (N = 5), according to the availability of the MLPA technique and the size of the CNV.

A better approximation of the CNV breakpoints could only be determinate in samples validated by CGH array.

## Results

### NGS variant quality and distribution

The coverage depth for the variants analyzed ranged from 20x to 361x, with a mean of 107.74x.

A total of 1109 variants in 425 genes were validated in 825 samples, including 872 SNVs, 214 indels, and 23 CNVs (Table 1). Of these, 79.6% were heterozygous (883), 17.85% homozygous (198), and 2.52% hemizygous (28).

**Table 1:** Clinical exome panels and variant distribution.

Panel	Samples	Variants analyzed	No. of genes with analyzed variants	SNVs	Indels	CNVs
TSO (4813 genes)	304	395	167	316	77	0
CES (4900 genes)	521	714	258	556	137	23
Total	825	1109	425	872	214	23

Variants were detected either from the analysis of the TSO (395 variants) or analysis of the CES samples (714 variants) (Table 1).

### NGS variant validation

**Figure 1:**
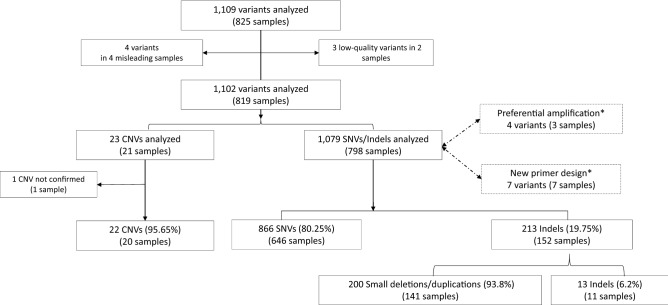
Validation process in the present study. (*) Samples with discordance encountered during the Sanger validation.

Misleading samples and low-quality variants were excluded from the NGS data validation. They represented 0.7% (6/825) of the total variants analyzed (Fig. 1). Of the remaining 819 samples considered for validation, a total of 1102 variants were analyzed: 1079 SNVs/Indels in 798 samples and 23 CNVs in 21 samples (Fig. 1).

High-quality SNVs and indel variants were checked by our team of experienced geneticists and manually visualized using the Integrative Genomics Browser tool before being further confirmed by Sanger sequencing. No false-positive SNVs (n = 866) or false-positive indel variants (n = 213) were detected, thus yielding a concordance of 100%.

However, even though they all resulted in true-positive cases of the NGS, we found 3 Sanger sequencing discrepancies (see Supplementary Figures S1–S3): (a) The heterozygous variant c.1819C > T (p.Arg607Cys) in the *NOTCH3* gene (NM_000435.2) was not detected by Sanger sequencing; (b) The heterozygous variant c.1256C > T (p.Pro419Leu) in the *TPRN* gene (NM_001128228) was detected as an homozygous variant by Sanger sequencing; (c) The heterozygous variant c.489C > G (p.Ser263Arg) in the *C1QTNF5* dominant gene (NM_015645.4) was detected as an homozygous variant by Sanger sequencing. None of these samples had rare variants within the primer regions or were located in a region with homopolymers or a region with pseudogenes. Because we were able to confirm both heterozygous variants with the redesign of the *NOTCH3* and *TPRN* primers, we concluded that a preferential amplification during Sanger sequencing was the most likely explanation. Regarding the *C1QTNF5* variant, the NGS analysis of the buccal cells, together with the familial segregation of the variant, also revealed a likely preferential amplification of the mutant allele by Sanger sequencing in this sample. However, the transcription of this *C1QTNF5* gene can be bicistronic—including the upstream membrane frizzled-related protein gene (MFRP)—or monocistronic, thus preventing us from ruling out the possibility that this peculiarity might somehow influence Sanger results.

Moreover, in at least 7 different samples out of the 170 total outsourced cases, the mutant allele was not detected during the first round of Sanger sequencing. A new primer design and a second round of Sanger sequencing were required to validate these samples.

Overall, we recorded problems in 10 Sanger sequences: 3 discrepancies were most likely due to preferential amplifications, and 7 discordances with the outsourced samples were due to problems with the specificity of primers or with the PCR conditions.

### CNV validation

During CNV validation, we found a heterozygous deletion of approximately 18 kb (chr12:88454782–88472858), presumably spanning exons 40 to 46 of the *CEP290* gene (NM_025114), that could not be further confirmed by a 400 k custom CGH-array including 12 probes covering the deleted region. MLPA was not available. Considering this as a false positive, we had a concordance of 95.65% (22/23) during the CNV validation (Table 2).

**Table 2:** CNVs analyzed in this study.

ID	Sex	CNV	Estimated size (Kb) by NGS	Zygosity	Minimal involved regions: Genes	Confirmed by
1	Male	Deletion	2.91	Het	Exons 8 to 10: *KCNH2* (NM_172057.2)	MLPA
2	Male	Deletion	3.31	Homo	Exons 7 and 8: *CLN3* (NM_001042432.1)	MLPA
3	Male	Deletion	4.32	Het	Complete gene (NM_019105): *TNXB* (NM_019105)	MLPA
4	Female	Duplication	10.65	Het	Complete gene: *FBN1* (NM_000138.4)	MLPA
5	Male	Deletion	12.56	Het	Exons 7 to 12: *LDLR* (NM_001195798.2)	MLPA
6	Female	Deletion	18.08	Het	Exons 40 to 46: *CEP290* (NM_025114.4)	Not confirmed by 400 k custom array
7	Female	Deletion	20.03	Het	Exons 11 to 16: *NIPBL* (NM_015384.5)	MLPA
8	Male	Deletion	104.90	Het	Exons 22 to 29: *USH2A* (NM_206933)	MLPA
9	Female	Deletion	109.28	Het	Exons 3 and 4: *MECP2* (NM_004992)	MLPA
10	Male	Deletion	117.39	Het	Exons 39 to 47: *USH2A* (NM_206933)	MLPA
11	Female	Deletion	206.66	Homo	Complete gene and exons 7 to 13 of: *STRC* (NM_153700) -*CASTPER2* (NM_172095.4)	MLPA
12	Male	Deletion	207.38	Het	Exon 1 to 9: *ITGB4* (NM_00213.5)	MLPA
13	Female	Deletion	224.40	Het	Exons 2 to 13: *PRPF31* (NM_015629.3)	MLPA
14	Male	Deletion	267.78	Hem	Exon 5: *RP2* (NM_006915.2)	MLPA
15	Female	Deletion	273.71	Het	Exons 3 to 10: *ANKRD11* (NM_013275.5)	a-CGH 60 k
16	Male	Deletion	449.14	Het	Exons 9 to 24: *DSP* (NM_004415.4)	MLPA
17	Male	Duplication	557.45	Het	chr10:102,957,798–103,443,479: *LBX1, BTRC, POLL, DPCD, FBXW4*	a-CGH 60 k
18	Female	Deletion	1982.87	Het	Complete gene: *ABCC6* (NM_001171)	MLPA
19	Male	Deletion	2050.09	Het	Complete gene: *STS* (NM_00351.5)	MLPA
20	Male	Duplication	2064.91	Het	chr17:34,816,424–36,207,539: *LHX1, ACACA*, *HNF1B*	a-CGH 60 k
21	Male	Deletion	3509.57	Het	chr22:19,438,161–19,956,753: UFD1L, GP1BB, TBX1, GNB1L, TXNRD2, COMT	a-CGH 60 k

*Het* heterozygous, *Homo* homozygous, *Hemi* hemizygous.

Of the remaining 22 validated CNVs in 20 samples, all but 2 were detected in different genes and all but 3 were heterozygous CNVs. Only 2 duplications were detected. The minimal and maximal CNV size detected by NGS in our samples was 2.91 Kb and 3.6 Mb respectively, with a medium of 207.02 Kb.

## Discussion

Validation data from various targeted panels have already been reported in several studies, with increasingly large sample sets^6–8^ (see summary Table 3). In 2013, Sikkema-Raddatz et al.^7^ evaluated 155 SNVs and 13 indels from 48 genes in 84 individuals. In 2015, Baudhuin et al.^8^ validated 797 SNVs and 122 unique indel variants from 117 captured genes in 84 patients, and, one year later in 2016, Mu et al.^6^ analyzed 6912 SNVs and 933 indels from 47 genes. Larger panels capturing 568 and 4813 genes have also been validated in 200 and 100 samples, respectively^4^, in which, excluding 4 low-quality variants and/or sample mix-ups, discrepancies were not observed. Interestingly, all of the abovementioned studies reported a concordance of 100% for high-quality NGS variants, concluding that Sanger sequencing is unnecessary in such cases.

**Table 3:** Summary of the NGS validation studies.

References	Targeted panel or exome analyzed	Analyzed patients	Number of Sanger analyzed variants	Variants quality conditions for which 100% concordance is obtained
Sikkema-Raddatz et al.^7^	48-genes targeted panel (SureSelect)	84	168 (155 SNVs, 13 indels)	Depth coverage > 30× Het ratio > 20%
Baudhuin et al.^8^	117-genes targeted panel(SureSelect)	84	919 (797 SNVs, 122 indels)	Depth coverage ≥ 100×, Variant quality score > 20 Flanking region mean base quality score > 15
Mu et al.^6^	49-genes targeted panel	20,000	7845 (6912 SNVs, 933 indels)	Depth coverage > 100× Het ratio > 40%
Nelson et al.^4^	568-genes inherited disorder panel (Sure Select) 4813- genesMendelian disease panel (Tru Sight One)	200 100	296 (255 SNVs, 41 indels)	PASS Depth coverage > 20× GQ score > 300 VAF 0.3–0.6 for het or > 0.9 or hom/hem
Beck et al.^10^	Clinical exome (SureSelect All Exon/TruSeq)	684	5800 variants	MPG score > 10
*Strom*	Clinical exome (SureSelect All Exon)	144	110 SNVs (94 unique SNVs)	Quality score ≥ Q500
Zheng et al.^12^	Whole exome (BGI)	342	855 (828 SNVs, 27 indels)	Depth coverage ≥ 35× Het ratio ≥ 35%
	1130 and 2181 targeted panel (SeqCap EZ)	3569	4777 (3684 SNVs, 1093 indels)	
	115 and 313-gene targeted panel (SeqCap EZ)	1279	1969 (1444 SNVs, 525 indels)	
		5190	7601 (5956 SNVs, 1645 indels)	
Current Study	167 (Tru Sight One) 258 (Clinical Exome Solution)	304 521	393 (316 SNVs, 77 indels) 693 (556 SNVs, 137 indels)	PASS QUAL ≥ 100 Depth coverage ≥ 20×, Variant fraction ≥ 20%
		825	1086 (872 SNVs, 214 indels)	

*VAF* variant allele frequency, *Het* heterozygous, *Hom* homozygous, *Hem* Hemizygous, *SNV* single nucleotide variants, *MPG* most probable genotype.

Validation data from exomes—in which lower-quality parameters are expected owing to the higher number of genes captured—have also been reported. Beck et al.^10^ evaluated over 5800 variants from 19 genes in 684 exomes, from which only 2 low-quality variants were discrepant. In 2014, Strom et al.^11^ validated 94 unique variants from 110 genes in 144 exomes, again with only 1 discordant low-quality variant. Recently, Zheng et al.^12^ extended this validation to 342 whole exomes and 4848 targeted panels, of which 3911 were analyzed using large Mendelian disease panels (1130 and 2182 genes). To our knowledge, this was the first study to validate exomes using BGI technology. Nevertheless, once again, all high-quality variants were further confirmed (100%).

In this study, we assessed the validation of the largest sample set of clinical exomes using Illumina technology. Remarkably, we found 3 discrepancies (3/789; 0.3%), all of which were resolved as false negatives of Sanger sequencing, thus demonstrating that this technique might not always be the best approach for confirming NGS variants, since, under specific conditions, the probability of detecting a false positive by NGS seems to be lower than that of detecting a false negative by Sanger sequencing^11^. Additionally, Sanger sequencing had to be repeated in 7 samples, leading to unnecessary consumption of time and money. Regardless of these drawbacks, we confirmed all of the 866 SNVs and 213 indel variants we had defined and filtered out as high-quality. Therefore, and consistent with these results, we decided to discontinue the validation of these variants, thus significantly reducing costs and turnaround times. Remarkably, this decision did not apply to variants that generated uncertainty for our geneticists by being associated either with the region where the variant was detected (i.e. homopolymers, pseudogenes) or with the nomenclature of the indel variant. Similarly, it did not apply to prenatal cases, where a double check is always performed, or to low-quality variants associated with the clinical suspicion, where validation is also recommended to decrease false-negative diagnosis. In this study, we do not evaluate the rate of false negative results in NGS. We have found 22 variants that, although didn’t strictly meet all the established quality parameters, were further confirmed by Sanger Sequencing for being pathogenic or likely pathogenic related-disease variants, supporting the importance of lowering the quality thresholds of these variants to decrease false-negative NGS results. Undoubtedly, the use of more sophisticated and machine-learning approaches would help to reduce these errors. Further studies are required to evaluate and establish the most appropriate quality threshold to obtain the best sensitivity of NGS in the clinical practice.

To our knowledge, there are no specific NGS guidelines that establish the quality parameters required for a variant to be considered high-quality, as this depends mainly on issues associated with the individual laboratory and on the NGS approach used^1^. For clinical exome panels, and based on our experience, we recommended the following: (1) FILTER = PASS, (2) QUAL ≥ 100, (3) depth coverage ≥ 20X, and (4) variant fraction ≥ 20%. However, laboratories should check and establish their thresholds before discontinuing the confirmation analysis.

In line with our results, Sanger sequencing is very useful as an internal quality control, as reflected by the 4 misleading samples we would not have detected otherwise. Unfortunately, even with high-quality measures, a laboratory cannot detect 100% of human errors. However, we believe these errors can be significantly minimized using proper controls such as the use of commercial sample tracking kits. Moreover, we propose the controls already implemented in our laboratory. First, the proband sample is always used as a positive control during segregation analysis. Because this is not possible for samples coming from external laboratories/hospitals, all external reported variants are further validated. Second, within the same sequencing run, we ensure that Sanger sequencing is performed in at least 4 different samples (at least 1 sample per pool). And third, the sex of each clinical exome analyzed is always checked prior to reporting, thus enabling us to detect at least 50% of errors. A counter to this measure is the detection (incidentally or not) of sex chromosome aneuploidy or a sex discrepancy due to an allogenic transplant in the patient. Urine or buccal cells, but not peripheral blood samples, would be the recommended samples to be analyzed in this last case.

To date, several studies have validated CNVs detected by targeted NGS panels^14–16^ and whole exomes^17^, with a wide variation in the false negatives and false positives reported in the analyses^16, 18^. Undoubtedly, their recent implementation in clinical practice has considerably increased our diagnostic yield, with the result that it is important to implement their detection during NGS. Here, we expand the validation of 22 CNVs detected by CES in 20 different samples. Our approach revealed wide variability in the CNV size detected within our cohort. Interestingly, one of the smallest CNVs detected (3.31 Kb) was homozygous, reflecting that a CNV is easily detected when the normal allele is not present. Interestingly, with the clinical exome approach, it is not possible to accurately establish the CNV detection limit of our NGS approach (CES), as this depends not only on the number of reads per sample, but also on the region and, specifically, on the number of probes within that specific region. In this study, it was not possible to accurately define the CNV breakpoints or size of most samples, nor was it possible to evaluate the incidence of false negatives within our samples.

We found a false-positive CNV in the *CEP290* gene, that is, a concordance of 95.65% (22/23); however, considering the small sample set analyzed, few conclusions can be drawn. Based on our experience, and despite the need for further validation, we believe that if a CNV is clearly related to the clinical suspicion, then it is very likely to be real. All suspected CNVs should be further validated with additional technology, which, in some cases, would enable us to determine specific breaking points. This is not always possible with exome sequencing technology. Change may come about with advances in genome sequencing techniques, as this robust approach makes it possible to detect specific CNVs^19^. However, many more large-scale validation studies are still needed.

## Conclusions

NGS is a robust technique for the detection of SNVs and indels that obviates the need for additional validation. Laboratories should establish their own quality thresholds before deciding to discontinue confirmation studies, and should implement internal quality controls in order to prevent human error and obtain reliable results. Our study enabled us to expand the validation data of 22 CNVs detected by NGS and highlight the importance of its implementation. Nevertheless, further validation of CNVs continues to be necessary.

## Supplementary Information

Supplementary Figures
