# Supplementary material for: Sanger sequencing is no longer always necessary based on a single-center validation of 1109 NGS variants in 825 clinical exomes
Source: Sci Rep. 2021 Mar 11;11:5697. doi: 10.1038/s41598-021-85182-w (PMC7952542; doi:10.1038/s41598-021-85182-w)

Sanger sequencing is no longer always necessary based on a single-center validation of 1,109 NGS variants in 825 clinical exomes

A. Arteche-López[1, 2], A. Ávila-Fernández[1], R. Romero [1], R. Riveiro Álvarez[1], M.A. López Martínez[1], A. Giménez Pardo[1], C. Vélez-Monsalve[1], J. Gallego Merlo[1], I. García Vara[1], B. Almoguera[1], A. Bustamante Aragonés[1], F. Blanco-Kelly[1], S. Tahsin Swafiri[1], E. Rodríguez Pinilla[1], P. Minguez[1], I. Lorda Sánchez[1], M.J. Trujillo Tiebas[1], C. Ayuso[1]

[1] Department of Genetics, Health Research Institute–Jimenez Diaz Foundation University Hospital (IIS-FJD), Madrid, Spain

[2] Department of Genetics, University Hospital 12 de Octubre, Madrid, Spain

<u>Supplementary Figure S1</u>

1A) IGV visualization of the heterozygous variant c.1819C>T (p.Arg607Cys) in the *NOTCH3* gene (NM_000435.2). 1B) Sanger chromatogram: the heterozygous variant is not detected. 1C) Sanger chromatogram: the heterozygous variant is only detected in the second round of amplification, after the redesign of primers. IGV=Integrative Genomics Viewer

1A

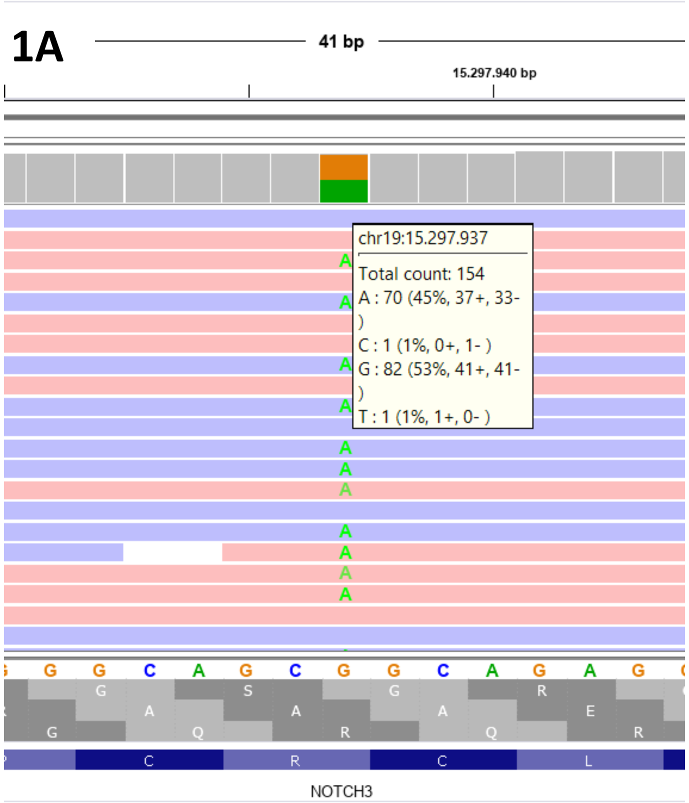

1B **Preferential amplification**

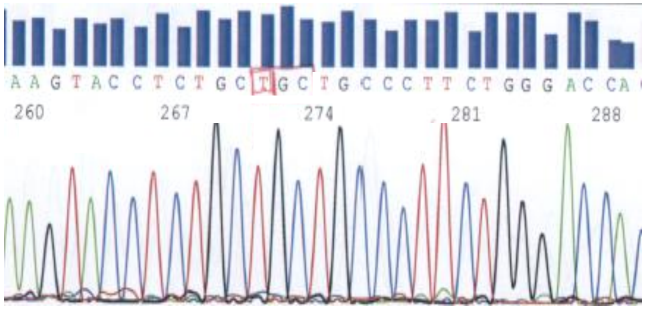

1C **Redesign of primers**

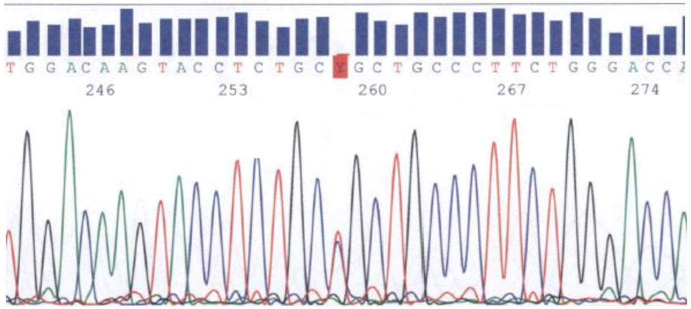

Supplementary Figure S2

2A) IGV visualization of the heterozygous variant c.1256C>T (p.Pro419Leu) in the *TPRN* gene (NM_001128228). 2) Sanger chromatogram: The variant is detected with both Forward (2B) and Reverse (2C), only after a redesign of primers. The sanger chromatogram showing the preferential amplification during the first round of PCR is not available. IGV=Integrative Genomics Viewer

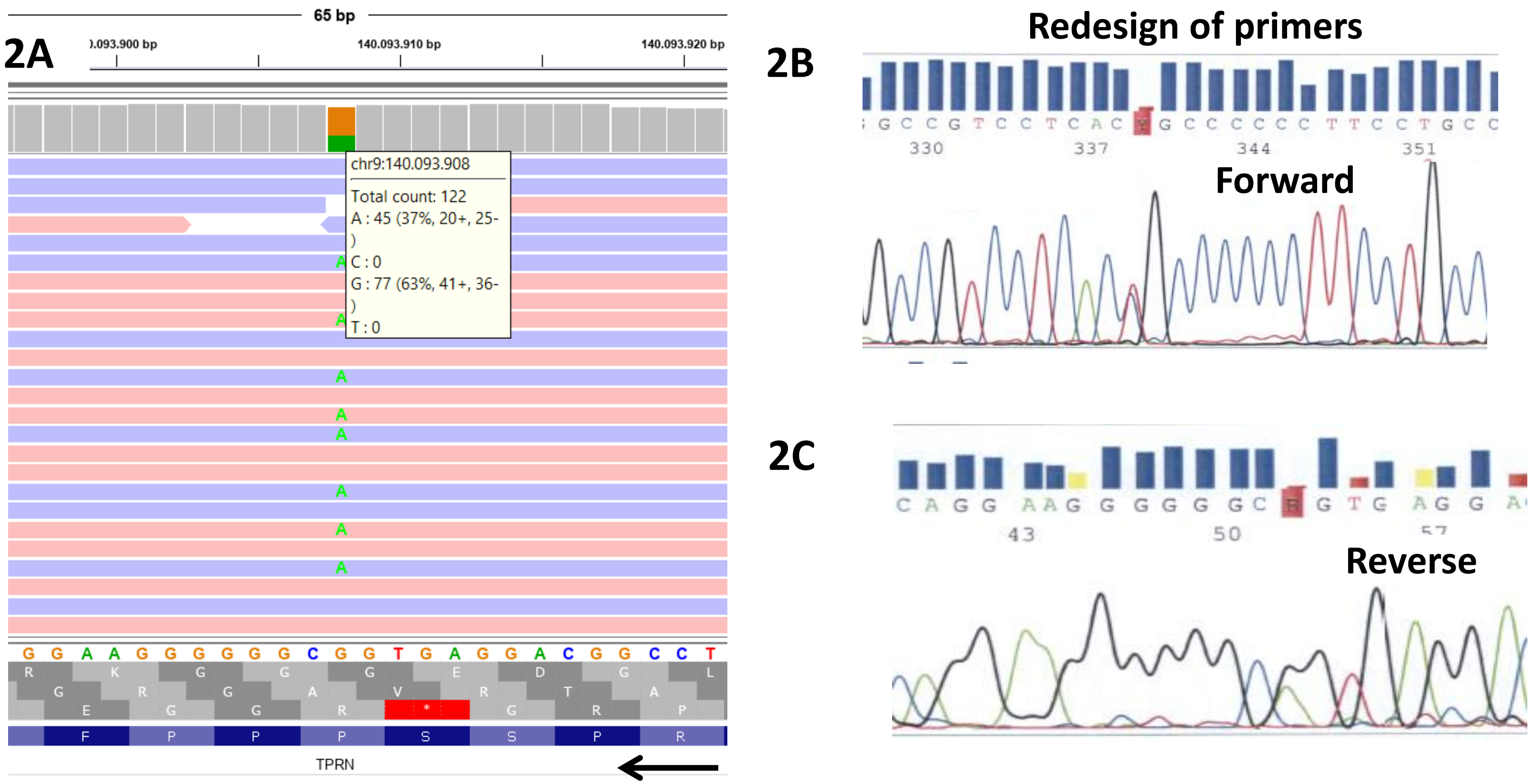

Supplementary Figure S3

3A) IGV visualization of the heterozygous variant c.489C>G (p.Ser263Arg) in the *C1QTNF5* gene (NM_015645.4). 3B) Sanger chromatogram of the variant in the Peripheral blood and Buccal cells (in bold), showing a preferential amplification of the mutant allele in the affected patient but not in the peripheral blood of the affected cousin. IGV=Integrative Genomics Viewer

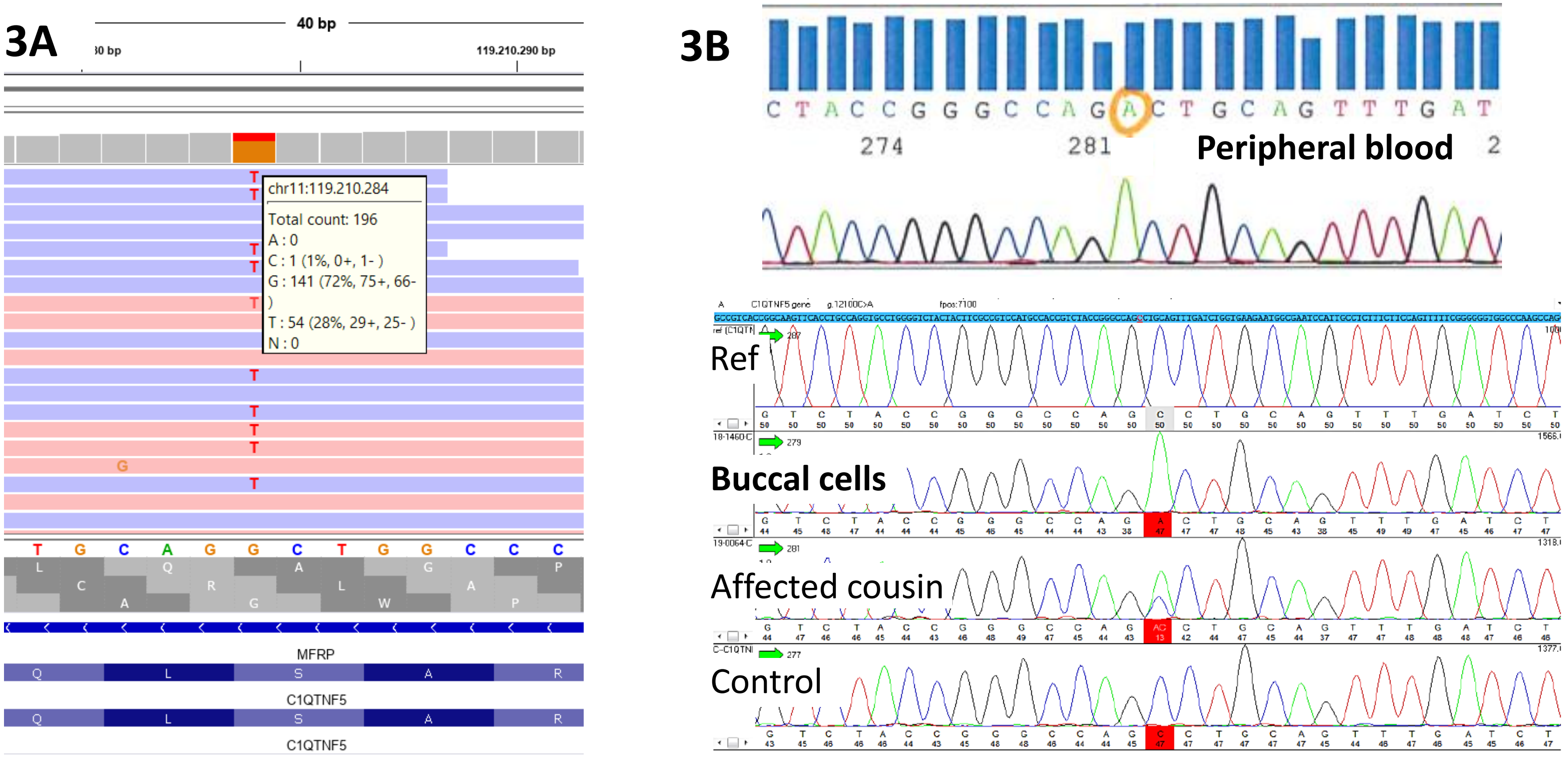

Supplement: Supplementary file 1 — Supplementary Figures [file 41598_2021_85182_MOESM1_ESM.pdf]
